# The Effects of Carbon Dots on Immune System Biomarkers, Using the Murine Macrophage Cell Line RAW 264.7 and Human Whole Blood Cell Cultures

**DOI:** 10.3390/nano8060388

**Published:** 2018-05-31

**Authors:** Kim Lategan, Jodi Fowler, Mohamed Bayati, Maria Fidalgo de Cortalezzi, Edmund Pool

**Affiliations:** 1 Department of Medical Bioscience, University of the Western Cape, Cape Town 7535, South Africa; 2917132@myuwc.ac.za (K.L.); 3242832@myuwc.ac.za (J.F.); 2 Department of Civil and Environmental Engineering, University of Missouri, Columbia, MO 65211, USA; mbb229@mail.missouri.edu (M.B.); fidalgom@missouri.edu (M.F.d.C.)

**Keywords:** carbon dots, cytotoxicity, induced inflammation

## Abstract

Carbon dots (CDs) are engineered nanoparticles that are used in a number of bioapplications such as bioimaging, drug delivery and theranostics. The effects of CDs on the immune system have not been evaluated. The effects of CDs on the immune system were assessed by using RAW 264.7 cells and whole blood cell cultures. RAW cells were exposed to CD concentrations under basal conditions. Whole blood cell cultures were exposed to CD concentrations under basal conditions or in the presence of the mitogens, lipopolysaccharide (LPS) or phytohaemmagglutinin (PHA). After exposure, a number of parameters were assessed, such as cell viability, biomarkers of inflammation, cytokine biomarkers of the acquired immune system and a proteome profile analysis. CDs were cytotoxic to RAW and whole blood cell cultures at 62.5, 250 and 500 μg/mL, respectively. Biomarkers associated with inflammation were induced by CD concentrations ≥250 and 500 μg/mL under basal conditions for both RAW and whole blood cell cultures, respectively. The humoral immune cytokine interleukin (IL)-10 was increased at 500 μg/mL CD under both basal and PHA activated whole blood cell culture conditions. Proteome analysis supported the inflammatory data as upregulated proteins identified are associated with inflammation. The upregulated proteins provide potential biomarkers of risk that can be assessed upon CD exposure.

## 1. Introduction

In 2004, carbon dots (CDs) were accidentally discovered when they were isolated from single-walled carbon nanotubes (SWCNTs) via gel electrophoresis [1]. CDs, also known as carbon quantum dots or carbon nanoparticles, are a relatively new fluorescent quasi spherical, zero-dimensional nanomaterial with a diameter less than 10 nm [2,3,4]. Recently, there has been an increase in CD popularity as they possess unique optical properties, biocompatibility, low toxicity, facile synthesis and aqueous stability [1,3,5]. These unique properties have allowed CDs to be used in bioapplications such as bioimaging, drug delivery and theranostic developments [1].

Due to these applications, human exposure is unavoidable. However, very few studies have evaluated the potential effects of CDs on cells and the immune system. In vivo and in vitro studies have mainly focused on the sensing and bioimaging of cells via the photoluminescent properties of CDs [6,7,8,9]. Only a few studies have reported on the toxicity of CDs, and these agree with the reports of their low toxicity. However, the toxicity is dependent on the functionalization of the CDs [10,11]. To our knowledge, no studies have reported on the effects of CDs on the immune system. Reports have been published on the effects of carbon nanotubes (CNTs) on the immune system [12,13,14]. In these studies, CNTs were reported to induce allergic airway inflammation, pulmonary inflammation, and inflammatory cytokines, interleukin 1β (IL-1β) and interleukin 6 (IL-6) under both in vivo and in vitro conditions.

This current study aimed to evaluate the effects of CDs on the murine macrophage cell line, RAW 264.7 and human whole blood cell cultures (WBCs). A number of parameters were assessed, which include cell viability, inflammatory biomarkers, cytokines of the acquired immune system and a proteome profile analysis.

## 2. Materials and Methods

### 2.1. Synthesis and Characterization of Carbon Dots (CDs)

**Figure A1:**
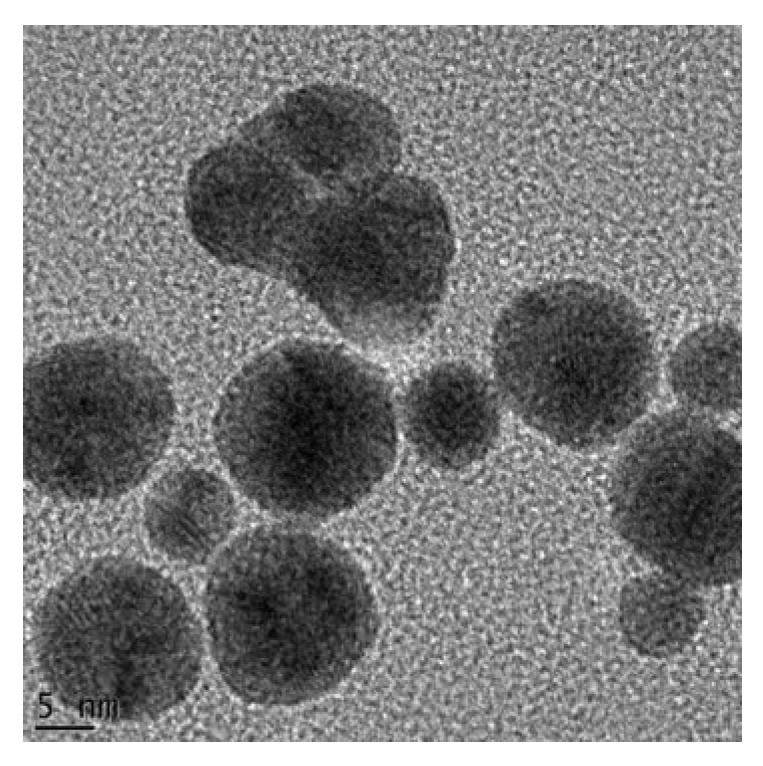
Visualization of carbon dots via transmission electron microscopy (TEM).

**Figure A2:**
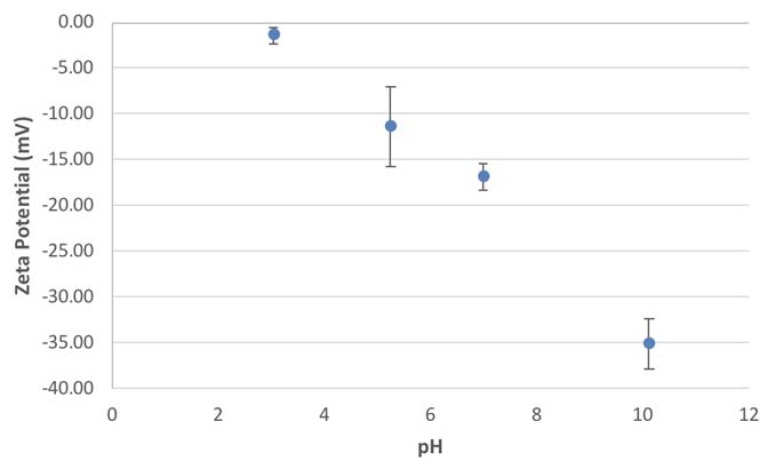
Zeta potential of carbon dots under varying pH levels, while maintaining a constant ion concentration of 10 mM NaCl.

The CDs were obtained from the University of Missouri where they were synthesized and characterized as previously published by Bayati et al. [2]. Briefly, transmission electron microscopy (TEM) (JEOL 1400, Peabody, MA, USA) (Figure A1) revealed an average size range of 1–3 nm and an average zeta potential of −16.83 ± 1.40 mV at a neutral pH in 1 mM sodium chloride (NaCl) (Figure A2 and Table A1).

### 2.2. Preparation of CD Stock Solutions

A 10 mg/mL stock solution of CDs in distilled water was prepared. The CDs were sonicated in short intermittent bursts on ice for approximately 5 min (QSonica, LLC. Misonix sonicators, XL-200 Series, Newtown, CT, USA). The CDs were sonicated prior to use in cultures.

### 2.3. RAW 264.7 Cells

#### 2.3.1. RAW 264.7 Macrophage Assay

The murine macrophage cell line, RAW 264.7, was obtained from the American Type Culture Collection (ATCC TIB-71, Manassas, VA, USA). The RAW 264.7 cells were cultured in Dulbecco’s modified Eagle’s medium (DMEM, Lonza, Cape Town, South Africa) supplemented with 10% heat inactivated fetal bovine serum (FBS, Hyclone, Little Chalfont, UK), glutamax (Sigma-Aldrich, St. Lousi, MO, USA), antibiotic/antimycotic (Sigma-Aldrich) and gentamicin (Sigma-Aldrich). The cells were incubated in a humidified atmosphere of 5% CO_2_ at 37 °C and the cells were sub-cultured every 2–3 days.

The RAW 264.7 cells (1 × 10^5^ cells/mL) were cultured in cell culture-treated 48-well plates and incubated in a humidified atmosphere of 5% CO_2_ at 37 °C for approximately 48 h until the cells reached 80–90% confluence. After the incubation period, the media was removed and replaced with media containing 2.5% FBS. The subsequent procedures occurred in serum free media. The cells were pre-exposed for 2 h to various concentrations of CDs. Thereafter, the cells were left unstimulated and a positive control was also present. The positive control was cells only stimulated by lipopolysaccharide (LPS) (1 µg/mL) without the presence of nanoparticle. The final concentration of FBS/well was 0.5%. Cultures were incubated overnight (~18 h) under standard tissue culture conditions. Culture supernatants were collected and used for nitric oxide (NO), interleukin 6 (IL-6), macrophage inflammatory protein 1α (MIP-1α), MIP-1β, MIP-2 and proteome profiling analysis.

#### 2.3.2. Cytotoxicity Assay

After the removal of the supernatants, cells were washed with Dulbecco’s phosphate buffered saline (DPBS) (Lonza), supplemented with glutamax and antibiotic/antimycotic solution. Cytotoxicity was measured by adding 150 µL of a 1/10 dilution of 2-(4-Iodophenyl)-3-(4-nitrophenyl)-5-(2,4-disulfophenyl)-2*H*-tetrazolium (WST-1, Roche, Basel, Switzerland) reagent in serum-free medium to each well. Metabolically active cells convert WST-1 reagent to a formazan that can be measured spectrophotometrically. Formazan formation was determined by reading the plate at 450 nm (Multiskan Ex, Thermo Electron Corporation, Waltham, MA, USA) immediately after WST-1 addition and again after an incubation period of 1 h at 37 °C. The increase in absorbance at 450 nm is proportional to formazan formation. The level of fomazan formed is directly proportional to cell viability.

#### 2.3.3. NO Determination

After the overnight incubation of the RAW 264.7 cells, the amount of nitrite that was produced by the cells was measured in the culture supernatant as an indication of NO production. The NO assay is based on the Griess reaction [15]. In this study, the NO assay is used to determine the amount of nitrite ion produced as a metabolite of NO. This was measured against a doubling dilution range of a 100 μM nitrite standard (Sigma-Aldrich). Nitrite standards or culture supernatant collected (100 µL) were mixed with 100 µL of Griess reagent (1:1 of 1% sulfanilamide and 0.1% naphtylethlemidimine-dihydrochloride in 2.5% phosphoric acid) (all reagents obtained from Sigma-Aldrich). Thereafter, the plate was incubated at room temperature for 15 min. The absorbance was read at 540 nm using a microplate reader (Multiskan Ex, Thermo Electron Corporation) and the amount of NO produced by the RAW cells quantified.

#### 2.3.4. Mouse IL-6 Double Antibody Sandwich (DAS) Enzyme Linked Immunosorbent Assay (ELISA)

The mouse IL-6 ELISA (e-Bioscience, Ready-Set-Go, Waltham, MA, USA) kits were used to measure IL-6 cytokine levels in the cell culture supernatants. The LPS-stimulated control was assayed at (1/40 *v*/*v*) while the negative control (not treated with LPS) was assayed at (1/5 *v*/*v*) in assay diluent. Assays were performed in 96-well Nunc maxisorb plates. The kit contained all the reagents for the assay and was performed as per the manufacturer’s instructions.

#### 2.3.5. Mouse MIPs (MIP-1α, MIP-1β and MIP-2) DAS ELISAs

Mouse MIP-1α, MIP-1β and MIP-2 ELISAs (R & D Systems, Minneapolis, MN, USA) were performed on the samples and the LPS-stimulated culture supernatants. The kits contained all the reagents required for the experiment and experiments were performed as per the manufacturer’s instructions. The samples were all diluted in reagent diluent, 1% human serum albumin (HSA) (*w*/*v*). The MIP-1α in unstimulated samples were assayed at 1/270 *v*/*v* and the LPS stimulated supernatant at 1/2000 *v*/*v* in diluent. For the MIP-1β ELISA, the unstimulated culture supernatants were assayed at 1/100 *v*/*v* while the LPS stimulated supernatants were assayed at 1/5000 *v*/*v* in diluent. The MIP-2 ELISA unstimulated supernatants were assayed at 1/20 *v*/*v* and the mitogen stimulated supernatant was at 1/500 *v*/*v* in assay diluent.

#### 2.3.6. Mouse Proteome Profiling Assay

A commercially available antibody array kit (Proteome Profiler, Mouse cytokine Array Panel A, R & D Systems) which was coated with 40 capturing antibodies in duplicate on a nitrocellulose membrane (dot blot) was used. The kit contained all the reagents for the assay and was performed as per the manufacturer’s instructions. This cytokine and chemokine antibody array was used to determine the effects of CD exposure on cytokine and chemokine synthesis by RAW 264.7 macrophage cells. The assay required 500 μL of cell culture supernatants (unstimulated containing 0 μg/mL CDs, LPS stimulated containing 0 μg/mL CDs, and unstimulated containing 500 μg/mL CDs). Membranes were subjected to an ultra-sensitive chromogenic 3,3′,5,5′-Tetramethylbenzidine (TMB) membrane substrate (Thermo Scientific, Waltham, MA, USA) to reveal sample–antibody complexes labeled with streptavidin-HRP. Photographs were taken of the blots after the exposure to the substrate.

#### 2.3.7. Quantification of Pixel Density for Cytokine and Chemokine Membranes

Membrane images were quantified using image processing and analysis Java software (version 1.6.0_24, Oracle Corporation, Redwood city, CA, USA), ImageJ (version 1.4.3.67, National Institutes of Health, Bethesda, MD, USA). Levels of cytokines and chemokines were expressed as a percentage of the reference spot. Microsoft Excel (Manufacturer name, city, state abbreviation if US or Canada, country) was used to calculate the percentage, which is expressed as mean ± standard deviation (SD).

### 2.4. Whole Blood Cell (WBC) Culture

#### 2.4.1. Blood Collection

Blood was collected by a doctor/nurse from healthy males not using any medication. The blood was collected using venipuncture directly into 3.2% sodium citrate vacuum tubes (Greiner bio-one, Kremsmunster, Austria). The blood was processed immediately. The whole blood cell cultures were performed under sterile conditions. Ethical clearance was obtained from the University of the Western Cape (Ethics No. 10/9/43). Informed consent was also obtained from the participant.

#### 2.4.2. Cell Culture

Human whole blood was diluted with RPMI-1640 media (Sigma-Aldrich) to give a 10% (*v*/*v*) blood in medium mixture. Blood was either left unstimulated or stimulated with LPS (Sigma-Aldrich) (0.1 μg/mL) or phytohaemmagglutinin (PHA) (Sigma-Aldrich) (1.6 μg/mL). Unstimulated or stimulated whole blood cell cultures were incubated overnight at 37 °C with high, intermediate and low concentrations of CDs in 24-well tissue culture treated plates (Nunc). A positive cytotoxicity control (medium containing 0.01% *v*/*v* Tween20 (Merck, Modderfontein, South Africa) was also present. After the incubation period, the culture supernatants were collected and assayed for cytotoxicity, cytokines and chemokines.

#### 2.4.3. Cytotoxicity Assay

Cytotoxicity was measured by monitoring lactate dehydrogenase (LDH) release by damaged cells. LDH activity was monitored spectrophotometrically using an LDH kit (LDH-cytotoxicity colourometric kit II, BioVision, Milpitas, CA, USA). The kit contained all the reagents required for the assay and assays were performed as per the manufacturer’s instructions.

#### 2.4.4. Cytokine Analysis using DAS ELISAs

Commercially available kits (e-Bioscience, Ready-Set-Go) were used to analyze the level of cytokine secretion from the whole blood cell cultures. The kits were used as per the manufacturer’s instructions and contained all the reagents to complete the assay. The unstimulated and LPS stimulated samples were analysed using a 1/10 dilution for the IL-6 assay. While the unstimulated and PHA stimulated samples were assayed neat for IL-10 and interferon gamma (IFNγ) analysis. The same protocol was used as previously described for the mouse cytokine ELISA.

#### 2.4.5. Human MIP-1β DAS ELISA

A human MIP-1β ELISA (R & D Systems) was performed on the unstimulated and LPS stimulated culture supernatants of the WBCs. The samples were diluted 1/10 in reagent diluent, 0.1% *v*/*v* bovine serum albumin (BSA) (Sigma). The same protocol was followed as for the mouse MIPs ELISAs.

#### 2.4.6. Human Proteome Profiling

A commercially available antibody array kit (Proteome Profiler, Human Cytokine Array Kit, R & D Systems) which was coated with 36 capturing antibodies in duplicate on a nitrocellulose membrane (dot blot) was used. The kit contained all the reagents for the assay and was performed as per the manufacturer’s instructions. This cytokine and chemokine antibody array was used to determine the effects of CD exposure on cytokine and chemokine secretion by WBCs. The assay required 500 μL of cell culture supernatants (unstimulated containing 0 μg/mL CDs, LPS stimulated containing 0 μg/mL CDs, and unstimulated containing 500 μg/mL CDs). The subsequent steps were carried out as described for the mouse cytokine and chemokine proteome profiling.

### 2.5. Statistical Analysis

All experiments were performed in triplicate and the data was calculated using Microsoft Excel. Data is presented as mean ± standard deviation (SD). One-way analysis of variance (ANOVA) using SigmaPlot 12.0 (Systat Software Inc., San Jose, CA, USA) was used to assess statistical differences with *p* < 0.01 being deemed significant.

## 3. Results

### 3.1. The Effects of CDs on RAW 264.7 Cells

#### 3.1.1. The Effects of CDs on the Viability of RAW 264.7 Cells

**Figure 1:**
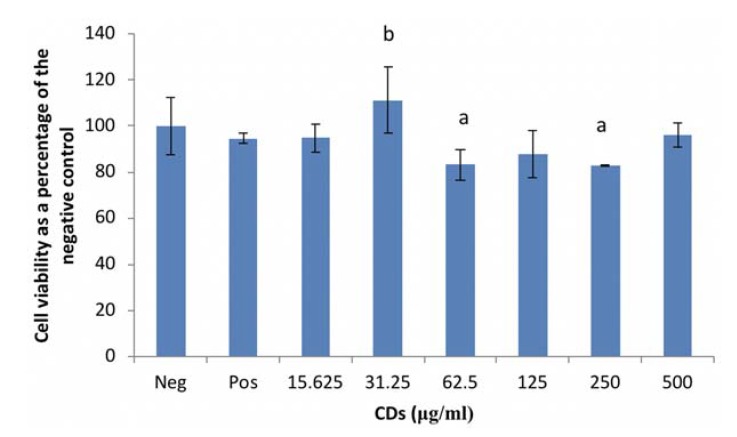
Cell viability of RAW 264.7 macrophage cells exposed to carbon dots (CDs). Data represents mean ± SD. with *n* = 9. Bars marked with letters indicate significant differences (*p* < 0.01). Significance. demarcated by: a—significantly different (*p* < 0.001) compared to 0 μg/mL CD control, b—significantly different (*p* < 0.001) compared to lipopolysaccharide (LPS)-stimulated 0 μg/mL CD control.

RAW cell viability, under basal conditions was significantly reduced (*p* < 0.001) by CD concentrations of 62.5 and 250 μg/mL compared to the culture control (Figure 1). However, viability was notably upregulated (*p* < 0.001) at 31.25 μg/mL CD compared to the positive control (LPS only). The other CD concentrations evaluated in this study had no effect on viability.

#### 3.1.2. The Effects of CDs on the Inflammatory Biomarker NO Using RAW 264.7 Cells

**Figure 2:**
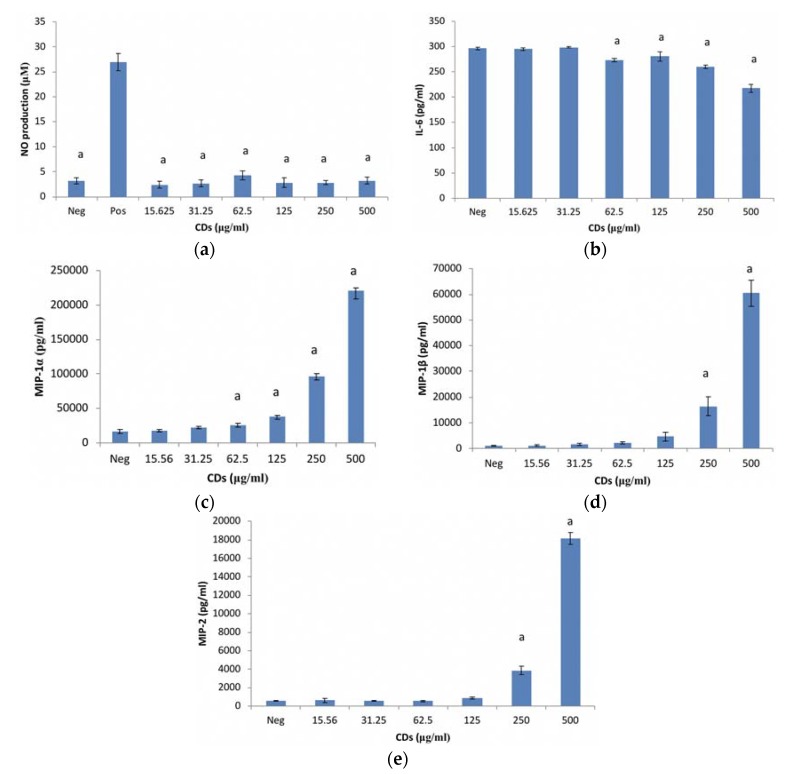
Murine macrophage, RAW 264.7 cells after treatment with CDs. Parameters assessed: (**a**) nitric oxide (NO) levels; (**b**) interleukin (IL)-6 production; (**c**) macrophage inflammatory protein (MIP)-1α; (**d**) MIP-1β and (**e**) MIP-2 production of unstimulated RAW 264.7 cell cultures exposed to CDs. Data represents mean ± SD with *n* = 9. Bars marked with letters indicate significant differences (*p* < 0.01). Significance demarcated by a: significantly different (*p* < 0.001), compared to the LPS stimulated 0 μg/mL CD control.

CDs at the concentrations tested, had no effect on NO production by unstimulated RAW 264.7 cells (Figure 2a). RAW cells treated with LPS in the absence of CDs were included as a positive control for inflammation.

#### 3.1.3. The Effects of CDs on the Inflammatory Biomarker IL-6 Using RAW 264.7 Cells

CD concentrations ≤ 31.25 μg/mL had no effect on IL-6 in an unstimulated environment (Figure 2b). However, CD concentrations ≥ 62.5 μg/mL significantly (*p* < 0.001) partially inhibited IL-6 synthesis from cells under basal conditions compared to the culture control. The positive control (56,748 ± 9591.8 pg/mL IL-6) is not included on the figure.

#### 3.1.4. The Effects of CDs on MIP-1α Using RAW 264.7 Cells

CD concentrations ≤ 31.25 μg/mL did not affect MIP-1α synthesis from RAW cells under unstimulated conditions (Figure 2c). However, CD concentrations ≥ 62.5 μg/mL significantly upregulated (*p* < 0.002) MIP-1α synthesis under basal conditions compared to the culture control. The positive control (LPS only) is not presented (1,637,093 ± 199,883.8 pg/mL MIP-1α) in Figure 2c.

#### 3.1.5. The Effects of CDs on MIP-1β Using RAW 264.7 Cells

MIP-1β synthesis was not affected by CD concentrations ≤ 125 μg/mL (Figure 2d). CD concentrations ≥ 250 μg/mL significantly upregulated (*p* < 0.001) MIP-1β synthesis from RAW cells under basal conditions compared to the culture control. The positive control, LPS (343,965 ± 52,044 pg/mL MIP-1β) is not presented on Figure 2d.

#### 3.1.6. The Effects of CDs on MIP-2 Using RAW 264.7 Cells

MIP-2 synthesis from RAW cells exposed to CD concentrations mimicked the MIP-1β data under basal conditions (Figure 2d). MIP-2 was unaffected by CD concentrations ≤ 125 μg/mL (Figure 2e). However, CD concentrations ≥ 250 μg/mL significantly upregulated (*p* < 0.001) MIP-2 synthesis from RAW cells under unstimulated conditions. The positive control (302,089 ± 68,868 pg/mL MIP-2) is not presented in Figure 2e.

### 3.2. The Effects of CDs on the Secretory Cytokine and Chemokine Profile of RAW 264.7 Cells

**Figure 3:**
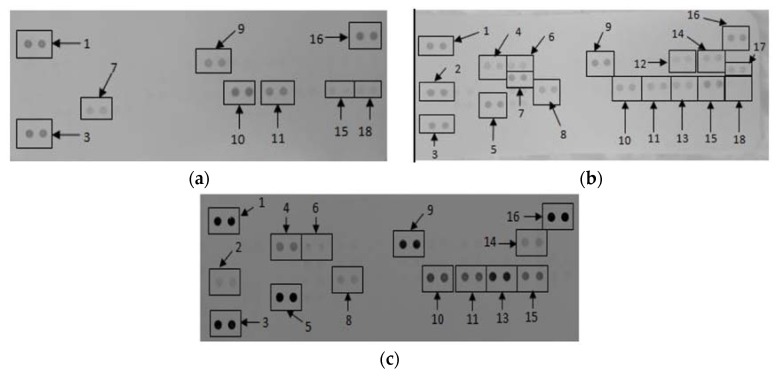
The effect of CDs on RAW 264.7 cells. Cells were incubated with (**a**) media only (negative control); (**b**) media in the presence of LPS and (**c**) 500 μg/mL CDs in the absence of a mitogen. Supernatants were probed using the proteome profiler array as described in methods. Cytokines/chemokines that were detected were allocated numbers: 1, 3, and 16 are reference spots; 2—IFNγ-inducible protein (IP)-10; 4—granulocyte-colony stimulating factor (G-CSF); 5—tumor necrosis factor (TNF)-α; 6—granulocyte–macrophage colony-stimulating factor (GM-CSF); 7—IL-6; 8—JE; 9—intracellular adhesion molecule (sICAM)-1; 10—MIP-1α; 11—MIP-1β; 12—IL-1β; 13—MIP-2; 14—interleukin 1 receptor antagonist (IL-1ra); 15—regulated on activation, normal T cell expressed and secreted (RANTES); 17—IL-27; 18—stromal cell-derived factor 1 (SDF-1).

Membranes exposed to culture supernatants of RAW 264.7 cells exposed to media only, media in the presence of a mitogen, and 500 μg/mL CDs in the absence of a mitogen allowed for the analysis of various cytokines and chemokines expressed by the cells upon exposure (Figure 3). Quantification of the membranes showed that RAW cells exposed to media containing LPS allowed the cells to synthesize certain proteins that were not synthesized by cells exposed to media only and 500 μg/mL CDs (Table 1). These proteins include macrophage colony-stimulating factor (M-CSF), interleukin 27 (IL-27) and interleukin 1 β (IL-β).

Similarly, certain proteins were significantly upregulated (*p* < 0.001) by cells exposed to 500 μg/mL CD compared to cells exposed to media only. These proteins include IFNγ-inducible protein 10 (IP-10), granulocyte-colony stimulating factor (G-CSF), tumor necrosis factor α (TNF-α), granulocyte–macrophage colony-stimulating factor (GM-CSF), IL-6, monocyte chemoattractant protein 1 (MCP-1)/JE gene of mouse fibroblasts, MIP-1α, MIP-1β, MIP-2, intracellular adhesion molecule 1 (ICAM-1) and interleukin 1 receptor antagonist (IL-1ra).

### 3.3. The Effects of CDs on WBCs

#### 3.3.1. The Effects of CDs on Viability of WBCs

**Figure 4:**
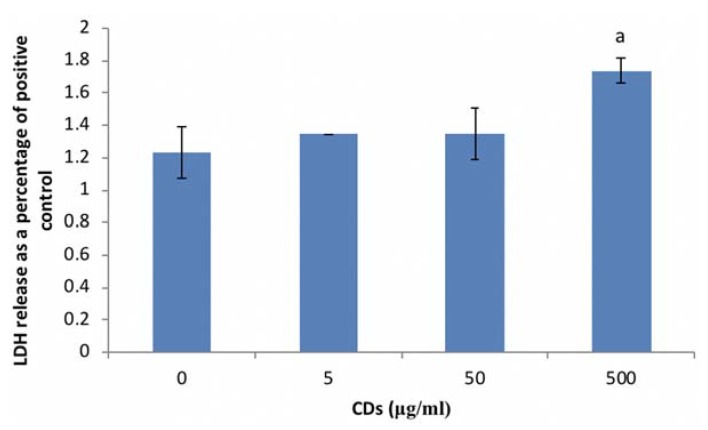
Cell viability of whole blood cells (WBCs) exposed to CDs. Data represents mean ± SD with *n* = 4. Bars marked with letters indicate significant difference (*p* < 0.01) to control. Significance demarcated by a: significantly different (*p* < 0.002) compared to 0 μg/mL CD control.

CD concentrations ≤ 50 μg/mL did not affect WBC viability (Figure 4). However, significant cytotoxicity (*p* < 0.002) was induced at the highest concentration of CD (500 μg/mL) screened.

#### 3.3.2. The Effects of CDs on the Inflammatory System Biomarker IL-6 Using WBCs

**Figure 5:**
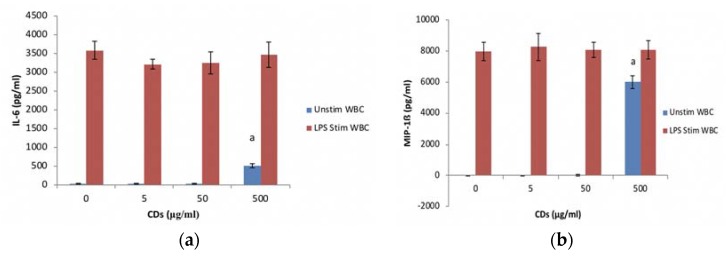
The inflammatory biomarker expression levels of whole blood cell cultures exposed to CDs. Nanoparticles (NPs) in the absence or presence of LPS: (**a**) IL-6 expression levels and (**b**) MIP-1β expression levels. Data represents mean ± SD with *n* = 4. Bars marked with letters indicate significant differences (*p* < 0.01). Significance demarcated by: a—significantly different compared (*p* < 0.002) to 0 μg/mL CD control.

CD concentrations ≤ 50 μg/mL did not affect IL-6 levels in cultures not stimulated with LPS (Figure 5a). However, at 500 μg/mL CD significantly induced the upregulation (*p* < 0.001) of IL-6 in unstimulated cultures. Cultures exposed to CD concentrations in the presence of LPS did not affect the synthesis of IL-6.

#### 3.3.3. The Effects of CDs on the Inflammatory Chemokine, MIP-1β Using WBCs

MIP-1β synthesis followed the same trend as the IL-6 data for both the unstimulated and stimulated cultures exposed to CDs. CD at concentrations tested had no effect on MIP-1β synthesis by LPS stimulated WBCs (Figure 5b). At concentrations ≤ 50 μg/mL, CD did not affect MIP-1β synthesis by unstimulated cells. However, CD at 500 μg/mL resulted in a significant (*p* < 0.001) stimulation of MIP-1β synthesis by unstimulated WBCs.

#### 3.3.4. The Effects of CDs on the Humoral Immune System Biomarker IL-10 Using WBCs

**Figure 6:**
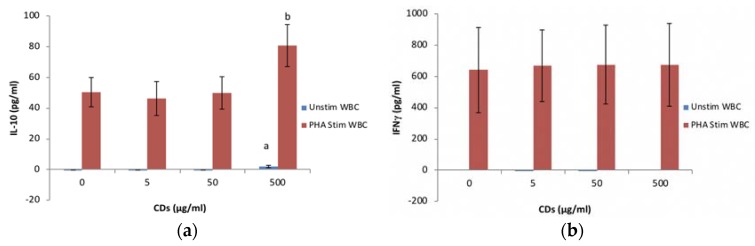
The acquired immune system biomarker expression levels of whole blood cell cultures exposed to CDs in the absence or presence of phytohaemmagglutinin (PHA): (**a**) IL-10 expression levels and (**b**) IFNγ expression levels. Data represents mean ± SD with *n* = 4. Significance demarcated by a: significantly different (*p* < 0.001) compared to 0 μg/mL CD control, b: significantly different (*p* < 0.008) compared to PHA stimulated 0 μg/mL CD control.

CD at 5 and 50 μg/mL did not affect the synthesis of IL-10 from unstimulated WBC cultures (Figure 6a). However, significant levels (*p* < 0.001) of IL-10 was detected in the 500 μg/mL CD unstimulated WBC culture compared to the negative control. PHA stimulated WBC at 5 and 50 μg/mL had no effect on IL-10 produced by WBCs compared to the positive control. However, at 500 μg/mL CD, the PHA stimulated WBC produced significantly more IL-10 compared to the positive control.

#### 3.3.5. The Effects of CDs on the Cell Mediated Immune System Biomarker IFNγ Using WBCs

None of the CD concentrations tested had an affect IFNγ synthesis by unstimulated PHA stimulated cultures (Figure 6b). IFNγ was not detected in unstimulated cultures.

### 3.4. The Effects of CDs on the Secretory Cytokine and Chemokine Profile of WBCs

**Figure 7:**
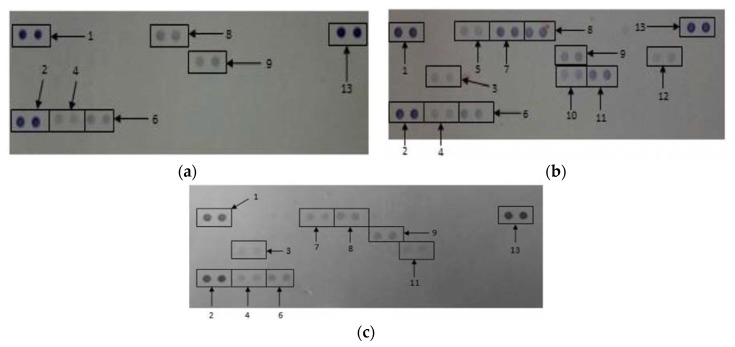
The effects of CDs on whole blood cells. Cells were incubated with (**a**) media only; (**b**) media and LPS; (**c**) 500 μg/mL CDs in the absence of LPS. Cytokines/chemokines that were detected were allocated numbers: 1, 2, and 13 are reference spots; 3—IL-1ra; 4—macrophage migration inhibitory factor (MIF); 5—MCP-1; 6—Serpin E1; 7—MIP-1α/β; 8—RANTES; 9—sICAM-1, 10—IL-6, 11—IL-8 and 12—IL-1β.

The proteome profile of WBCs exposed to media only and media in the presences of LPS and 500 μg/mL CD in media only revealed the synthesis or inhibition of certain proteins (Figure 7).

Quantification of the membranes showed that certain proteins were significantly upregulated (*p* < 0.001) in supernatants at WBCs incubated in the presence of 500 μg/mL CD compared to the negative control (media only) (Table 2). These proteins include IL-1ra, macrophage migration inhibitory factor (MIF), MIP-1α/β and IL-8. Cytokines and chemokines that were prominently down regulated compared to the positive control include IL-1ra, MCP-1, MIP-1α/β, regulated on activation, normal T cell expressed and secreted (RANTES), IL-6, IL-8 and IL-1β.

## 4. Discussion

In recent years, CDs has attracted lots of interest due to their many bioapplications, such as bioimaging, drug delivery and theranostics [1]. However, no studies have evaluated the effects these CDs have on the immune system.

Exposing RAW cells to CD concentrations of 62.5 and 250 μg/mL reduced cell viability by less than 20%. Viability was also reduced in WBCs exposed to the highest concentration of CDs (500 μg/mL). Due to the results seen in WBCs, future studies will investigate the effects of CD on WBC viability at concentrations between 50 and 500 μg/mL. The results obtained in this study for both RAW cells and WBCs supports previous reports of CDs having low toxicity levels [3,5]. Havrdova et al. (2016) found that pristine CDs only induced morphological changes in cells at concentrations >200 μg/mL [10]. They also found that pristine CDs stimulated proliferation at very low concentrations, ranging from 5 to 50 μg/mL, and thereafter a decrease in viability, with an increase in concentration. This trend was followed in this study as there was an increase in cell viability of the RAW cells at 31.25 μg/mL and a reduction in viability at 62.5 and 250 μg/mL. This could be attributed to a high level of reactive oxygen species leading to uncontrolled proliferation in the G2/M phase of the cell cycle.

Studies have mainly monitored the bioimaging of cells using CDs [6,7,8]. Wang et al. (2011) evaluated the cytotoxic effects of CDs on two different cell types, HT-29 and MCF-7 [16]: cytotoxicity was induced in both cell lines, although cytotoxicity was also dependent on the functionalization of the CDs. However, Yang et al. (2009) had contradictory results to what was found by Wang et al. (2011) as they reported no effects on all parameters monitored, using the same cell lines, but only poly(ethylene glycol-amine) (PEG) functionalization of CDs [16,17]. The effects of SWCNTs on RAW cells was evaluated and it was found that CDs induced oxidative stress [18]. However, Crouzier et al. (2010) found that carbon nanotubes reduced oxidative stress in the lungs of mice [19].

Under basal conditions, the RAW cell inflammatory marker IL-6 was partially inhibited by CD concentrations ≥ 62.5 μg/mL. However, NO remained unaffected by CD exposure. Contradictory to the RAW cell inflammatory markers, the MIP chemokines were significantly upregulated by CD exposure at concentrations ≥ 250 μg/mL. A similar trend was seen with the WBCs, where the highest concentration assessed (500 μg/mL) induced IL-6 and MIP-1β in unstimulated cultures. No studies have been conducted on the effects of CDs on the immune system. In vitro monocyte cultures exposed to carbon nanotubes induced the formation of inflammatory genes IL-6 and IL-1β [16]. Kayat et al. (2011) and Crouzier et al. (2010) also found that carbon nanotubes induced inflammatory markers [13,19]. Another study conducted by Murray et al. (2009) found that SWCNTs induced pro-inflammatory cytokines such as IL-6, MCP-1 and TNF-α in dermal cells [20]. Zhang et al. (2012) also found that RAW cells exposed to multi-wall carbon nanotubes (MWCNTs) induced inflammatory proteins [21]. The induction of inflammatory markers was supported by the proteome profile analysis of both RAW and WBCs. The inflammatory proteins induced included TNF-α, MIPs, MCP-1, IL-6, IL-8, IL-1ra and IL-1β. However, Liu et al. (2012) found that CDs did not affect inflammatory genes such as IL-6 and TNF-α [22].

The humoral immune response cytokine IL-10 was upregulated at 500 μg/mL CD in both unstimulated and PHA stimulated cultures, whereas the cell mediated immune response cytokine IFNγ remained unaffected under both conditions. The induction of IL-10 in unstimulated conditions would provoke a humoral immune response, when one is not required and upregulated such a response under stimulated conditions. This could lead to hypersensitivity and autoimmunity reactions [23].

## 5. Conclusions

This study clearly demonstrates that CDs are cytotoxic at high concentrations, although the levels of cytotoxicity are low. CDs also induce inflammation under basal conditions at relatively high concentrations, supported by the proteome analysis. The proteome analysis revealed potential biomarkers to be assessed upon CD exposure. These include MIP-1α, MIP-1β, MIP-2, TNF-α, IL-8 and SDF-1. The humoral immune response is also modulated at high levels of CDs. This stimulation of immune responses could lead to hypersensitivity and autoimmunity [23]. The current study provides an important insight into the effects of CDs on the immune system as these nanoparticles are geared to be used in vivo for a number of bioapplications.

## Figures and Tables

**Table 1:** Quantification of cytokines and chemokines secreted by RAW 264.7 cultures not stimulated with LPS after treatment with medium only, medium containing LPS or medium containing 500 μg/mL carbon dots (CDs). Membranes subjected to chromogenic exposure. Data is represented as mean ± SD. Significance indicated by ^a^ CD at 500 μg/mL significantly different compared to negative control (*p* < 0.001), ^b^ CD at 500 μg/mL significantly different compared to the positive control (*p* < 0.001).

Cytokines and Chemokines	Positive Control	Negative Control	500 μg/mL CDs
Reference Spot	100 ± 8.62	100 ± 10.49	100 ± 15.19
IP-10	114.74 ± 5.24	0 ± 0	8.20 ± 0.11 ^a,b^
G-CSF	58.27 ± 2.55	0 ± 0	26.76 ± 0.51 ^a,b^
TNF-α	102.01 ± 5.91	17.96 ± 0.13	101.00 ± 0.60 ^a^
GM-CSF	47.32 ± 3.02	0 ± 0	3.72 ± 0.03 ^a,b^
IL-6	91.94 ± 3.60	0 ± 0	0 ± 0 ^b^
JE	78.97 ± 4.48	0 ± 0	10.47 ± 0.01 ^a,b^
sICAM	109.05 ± 6.00	34.76 ± 0.02	78.65 ± 0.98 ^a,b^
MIP-1α	72.87 ± 4.22	57.00 ± 1.94	64.87 ± 1.87 ^a,b^
MIP-1β	62.77 ± 3.35	37.68 ± 0.33	45.40 ± 0.81 ^a,b^
MIP-2	55.51 ± 2.75	0 ± 0	76.63 ± 0.06 ^a,b^
RANTES	100.57 ± 4.42	17.93 ± 0.83	36.09 ± 0.78 ^a,b^
SDF-1	16.41 ± 1.61	21.75 ± 1.01	0 ± 0 ^a,b^
IL-27	54.97 ± 4.05	0 ± 0	0 ± 0 ^b^
IL-1ra	55.84 ± 1.66	0 ± 0	14.96 ± 0.21 ^a,b^
IL-1β	39.16 ± 3.31	0 ± 0	0 ± 0 ^b^

**Table 2:** Quantification of cytokines and chemokines secreted by Whole Blood Cells (WBCs) not stimulated with LPS after treatment with medium only (negative control), medium containing LPS (positive control) or medium containing 500 μg/mL CDs. Membranes were subjected to chromogenic exposure. Data is represented as mean ± SD. Significance indicated by ^a^ CDs at 500 μg/mL significantly different (*p* < 0.01 compared to negative control, ^b^ CDs at 500 μg/mL significantly different (*p* < 0.001) compared to the positive control.

Cytokines and Chemokines	Positive Control	Negative Control	500 μg/mL CDs
Reference Spot	100 ± 7.28	100 ± 5.57	100 ± 11.35
IL-1ra	16.92 ± 1.46	0 ± 0	12.57 ± 1.27 ^a,b^
MIF	17.56 ± 1.51	10.57 ± 1.91	20.41 ± 4.00 ^a^
MCP-1	26.26 ± 2.54	0 ± 0	0 ± 0 ^b^
Serpin E1	32.40 ± 2.49	24.26 ± 2.26	31.81 ± 4.38
MIP-1α/β	70.22 ± 4.11	0 ± 0	25.84 ± 2.17 ^a,b^
RANTES	62.94 ± 2.55	34.28 ± 1.37	32.95 ± 2.75 ^b^
sICAM	37.15 ± 0.59	35.98 ± 2.19	32.38 ± 4.52
IL-6	29.31 ± 4.81	0 ± 0	0 ± 0 ^b^
IL-8	50.27 ± 8.41	0 ± 0	13.56 ± 2.53 ^a,b^
IL-1β	20.29 ± 0.99	0 ± 0	0 ± 0 ^b^

## Appendix A

**Table A1:** Average size of carbon dots, under varying pH levels, while maintaining a constant ion concentration of 10 mM NaCl.

pH	Average	Standard Deviation
3.04	−1.42	0.87
5.25	−11.40	4.35
6.99	−16.83	1.40
10.12	−35.07	2.74
